# Dosimetric evaluation of synthetic CT generated with GANs for MRI‐only proton therapy treatment planning of brain tumors

**DOI:** 10.1002/acm2.12856

**Published:** 2020-03-26

**Authors:** Samaneh Kazemifar, Ana M. Barragán Montero, Kevin Souris, Sara T. Rivas, Robert Timmerman, Yang K. Park, Steve Jiang, Xavier Geets, Edmond Sterpin, Amir Owrangi

**Affiliations:** ^1^ Medical Artificial Intelligence and Automation (MAIA) Laboratory Department of Radiation Oncology University of Texas Southwestern Medical Center Dallas TX USA; ^2^ Institut de Recherche Expérimentale et Clinique Center of Molecular Imaging, Radiotherapy and Oncology (MIRO) Université catholique de Louvain Brussels Belgium; ^3^ Department of Oncology Laboratory of Experimental Radiotherapy KULeuven Leuven Belgium; ^4^ Department of Radiation Oncology Cliniques universitaires Saint‐Luc Brussels Belgium

**Keywords:** artificial intelligence, brain MRI, deep learning, GAN structure, proton therapy, synthetic CT

## Abstract

**Purpose:**

The purpose of this study was to address the dosimetric accuracy of synthetic computed tomography (sCT) images of patients with brain tumor generated using a modified generative adversarial network (GAN) method, for their use in magnetic resonance imaging (MRI)‐only treatment planning for proton therapy.

**Methods:**

Dose volume histogram (DVH) analysis was performed on CT and sCT images of patients with brain tumor for plans generated for intensity‐modulated proton therapy (IMPT). All plans were robustly optimized using a commercially available treatment planning system (RayStation, from RaySearch Laboratories) and standard robust parameters reported in the literature. The IMPT plan was then used to compute the dose on CT and sCT images for dosimetric comparison, using RayStation analytical (pencil beam) dose algorithm. We used a second, independent Monte Carlo dose calculation engine to recompute the dose on both CT and sCT images to ensure a proper analysis of the dosimetric accuracy of the sCT images.

**Results:**

The results extracted from RayStation showed excellent agreement for most DVH metrics computed on the CT and sCT for the nominal case, with a mean absolute difference below 0.5% (0.3 Gy) of the prescription dose for the clinical target volume (CTV) and below 2% (1.2 Gy) for the organs at risk (OARs) considered. This demonstrates a high dosimetric accuracy for the generated sCT images, especially in the target volume. The metrics obtained from the Monte Carlo doses mostly agreed with the values extracted from RayStation for the nominal and worst‐case scenarios (mean difference below 3%).

**Conclusions:**

This work demonstrated the feasibility of using sCT generated with a GAN‐based deep learning method for MRI‐only treatment planning of patients with brain tumor in intensity‐modulated proton therapy.

## 1 INTRODUCTION

Magnetic resonance imaging (MRI) is often used in radiation therapy to accurately contour the clinical target volume (CTV) and organs at risk (OARs) because of its superior soft tissue contrast compared with computed tomography (CT) images. The use of MRI images is especially crucial in treatment sites in the abdomen and brain, where the tumor volume is mainly surrounded by soft tissue. However, CT images are still required to retrieve information about the physical quantities needed for dose calculation, that is, electron density for radiation therapy with photons and stopping powers for ion therapy.^1^ Therefore, the current treatment planning workflow for these sites relies on contouring the target and OARs on MRI, then transferring the contours to CT via image registration. Magnetic resonance imaging‐CT co‐registration introduces geometrical uncertainties of ~2 mm for the brain^2^, ^3^ and 2–3 mm for prostate and gynecological patients.^4^ Importantly, these errors are systematic, persist throughout treatment, shift high‐dose regions away from the target,^5^ and may lead to a geometric miss that compromises tumor control. This problem has recently led to the concept of MRI‐only–based treatment planning, where pseudo or synthetic CT (sCT) images for dose calculation are generated directly from the MRI scan. Magnetic resonance imaging‐only treatment planning would also reduce radiation dose, imaging time, and hospital resources.^6^ Magnetic resonance imaging‐only treatment planning is, then, an attractive concept that is gaining popularity.^7^ However, accurately generating Hounsfield unit (HU) maps from MRI images is not straightforward.

The conventional methods proposed in the literature for automatically generating sCT images can be divided into four categories: bulk density methods, voxel‐based or tissue segmentation‐based methods, single‐ or multi‐atlas registration with fusion algorithms, and hybrid approaches that combine both atlas‐ and machine learning‐based approaches.^8^, ^9^ The accuracy of these methods has improved with time, but they still suffer from several limitations. Voxel‐ or tissue segmentation‐based methods either require the acquisition of multiple MRI sequences, which result in a longer scanning time, or they use nonstandard sequences seldom available in clinical routines, such as ultrashort echo time (UTE), to segment bone and air regions.^10^ Atlas‐based methods often fail to handle atypical patient anatomy and may cause intersubject registration errors.^11^, ^12^ It has been demonstrated that using multiple atlases improves the results, but the optimal number of atlases remains a question to address.^8^, ^13^ The combination of atlas‐based registration and machine learning‐based methods has demonstrated superior accuracy,^14^, ^15^ but these methods largely depend on handcrafted features, which present a twofold weakness: first, defining these features requires human intervention, and second, it is still uncertain which features have the greatest impact on the model's accuracy. To overcome these problems, deep learning methods have recently been proposed, because they completely eliminate dependence on handcrafted features by allowing the deep network to learn its own optimal features to accurately generate sCT images. Several groups have reported a lower HU error between synthetic and real CT images with deep learning‐based methods than with conventional methods, such as atlas‐based methods.^14^ In addition, deep learning‐based methods showed excellent dosimetric accuracy for treatment plans based on sCT images generated for brain^16^ and prostate patients^17^ treated with conventional radiation therapy with photons.

However, these small errors in the HU maps generated may still lead to large dosimetric differences for proton therapy treatments because of the proton range's high sensitivity to the tissue traversed along the beam path.^18^, ^19^ The literature is sparse regarding the dosimetric evaluation of sCT generation methods for proton therapy,^20^, ^21^, ^22^, ^23^ but a couple of groups that analyzed the performance of conventional methods based on tissue segmentation reported, indeed, the need to manually pre‐ or post‐process the pseudo HU values to minimize proton range differences and ensure reasonable dosimetric accuracy. For instance, Koivula et al.^20^ segmented bone regions before assigning the corresponding HU, while Maspero et al.^21^ manually inserted air cavities within the body contour as found in the CT images to minimize interscan differences (at different time points). Using the newly developed deep learning methods mentioned above could help to achieve higher accuracy while removing any manual operations. In the last year, several groups have started to investigate the application of deep learning for sCT generation, achieving very promising results.^24^, ^25^, ^26^, ^27^ In addition, they analyzed the dosimetric accuracy of the generated sCT for single field uniform dose (SFUD) and conventional PTV optimization. But to our knowledge, a proper dosimetric evaluation of these methods for fully intensity‐modulated proton therapy (IMPT) with robust optimization has not been performed yet. This article aims to address this issue by analyzing the performance of a deep learning sCT generation method based on generative adversarial networks (GANs) for IMPT treatment planning. Specifically, we focus on treatment plans for brain patients that have been robustly optimized using a commercially available treatment planning system (RayStation, from RaySearch Laboratories) and standard robust parameters^28^, ^29^ reported in the literature (3 mm for the systematic setup error and 3% for the range uncertainty). Robust optimization is the state of the art for treatment planning in proton therapy, and it might help to mitigate the small HU errors in the generated sCT images. However, robustness must be properly evaluated to analyze dosimetric accuracy in all possible scenarios, accounting for both conventional delivery errors and the uncertainties inherent in the sCT generation algorithm, which is crucial to ensure correct treatment outcomes in proton therapy. For this purpose, although the plans were optimized using the analytical dose algorithm embedded in RayStation, we used an independent Monte Carlo dose engine for the final dose recalculation and robustness evaluation.

## 2 MATERIALS AND METHODS

### 2.1 Image acquisition

We analyzed CT and MRI images from patients who had undergone conventional radiotherapy for brain tumors. Tumor sizes varied between 1.1 and 42.4 cm^3^. The images were collected at the ‐ University of Texas Southwestern Medical Center‐ as part of the standard treatment protocol. Patients underwent both CT and MRI scanning for radiotherapy treatment planning. All CT images were acquired in the Department of Radiation Oncology using a 16‐slice CT (Phillips Big Bore scanner, Royal Philips Electronics, Eindhoven, The Netherlands), 120 kV, exposure time = 900 ms, and 180 mA. CT images were acquired with a 512 × 512 matrix and 1.5 mm slice thickness (voxel size 0.68 × 0.68 × 1.50 mm^3^). The MRI images were acquired using a 1.5 T magnetic field strength and a post‐gadolinium two‐dimensional (2D) T_1_‐weighted spin echo sequence with TE/TR = 15/3500 ms, a 512 × 512 matrix, and an average voxel size of 0.65 × 0.65 × 1.5 mm^3^. The CT and MRI were acquired on the same day/week depending on the availability of the scanner.

### 2.2 Generative adversarial networks (GANs)

Generative adversarial networks^30^ are a class of deep machine learning algorithms used in unsupervised learning and are composed of two convolutional neural networks (CNN) that compete against each other: one CNN generates sCT candidates (*generator*), while the other CNN evaluates them by comparing them with real CT images (*discriminator*). This process is repeated until the discriminator can no longer distinguish between the real CT and the sCT, which indicates that the generator has learned to accurately transform MRI to CT images. This work applied the concept of conditional GAN^31^ but modified the original model to improve its performance for our particular application. First, we used U‐Net^32^ with mutual information (MI) as the loss function to overcome difficulties in MRI‐to‐CT registration, and second, we used several convolutional layers and several fully connected layers with rectified linear unit (ReLU)^33^ and binary cross entropy as the activation/loss functions in the discriminator network. In the following paragraphs, we describe more detail of both the generator and the discriminator components of the conditional GAN model.

#### 2.2.1 Generator

**Fig. 1:**
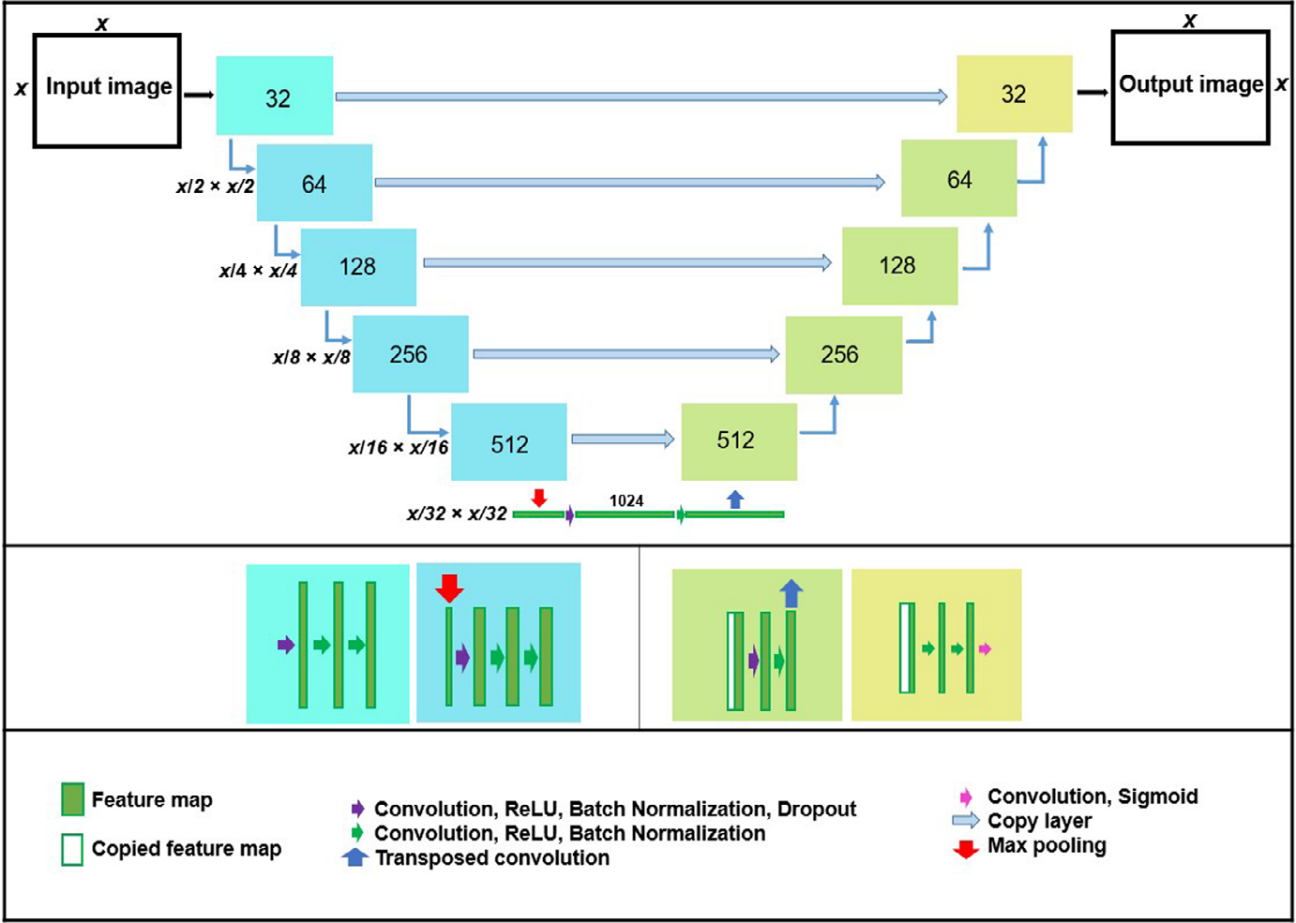
The structure of the generator with the details of convolutional and de‐convolutional layers.

Our model uses a 2D U‐Net as the generator network, which directly learns a mapping function to convert a 2D grayscale image to its corresponding 2D sCT image. Our generator network contains blocks of convolutional 2D layers with variable filter sizes, but the same kernel sizes and activation functions, except the last layer. The structure of our U‐Net generator model is illustrated in Fig. 1. On the left side of the U‐Net structure, the low‐level feature maps are downsampled to high‐level feature maps using a max pooling layer. Therefore, we used three 3 × 3 convolutional layers,^34^, ^35^ each followed by an ReLU (activation function), and one max pooling operation. On the right side of the U‐Net structure, the high‐level feature maps and low‐level feature maps are fed to the upsampling step using the transposed convolutional layer to construct the predicted image. Therefore, we used a 2 × 2 transposed convolutional layer followed by a concatenate layer and added two 3 × 3 convolutional layers with an ReLU activation function. In addition, a batch normalization layer was added to each 3 × 3 convolutional layer, and a dropout layer was added to one 3 × 3 convolutional layer. In the final layer, we used a 1 × 1 convolutional layer with filter size (1) and a sigmoid activation function. The generator's loss function was MI, using an Adam optimizer, of learning rate = 0.0002, beta_1 = 0.5 (exponential decay rates for the moment estimates^36^).

#### 2.2.2 Mutual information cost function

We defined the custom loss function “mutual information” between CT and sCT of the generator using Keras package. MI measures the “amount of information” of one variable when another variable is known. Maximizing MI is equivalent to minimizing the joint entropy (joint histogram). The MI between our two variables, the real CT ( $x_{i}$ ) and the generated sCT ( $G\left(y_{i}\right)$ , with $y_{i}$ as the MRI), is expressed as:

$$MI\left(x_{i},G\left(y_{i}\right)\right)=\sum_{x_{i},G\left(y_{i}\right)}p\left(x_{i},G\left(y_{i}\right)\right)log\frac{p\left(x_{i},G\left(y_{i}\right)\right)}{p\left(x_{i}\right)p\left(G\left(y_{i}\right)\right)}=H\left(x_{i}\right)+H\left(G\left(y_{i}\right)\right)-H\left(x_{i},G\left(y_{i}\right)\right)$$

where $p\left(x_{i},G\left(y_{i}\right)\right)$ is the joint distribution, and $p\left(x_{i}\right)$ and $p\left(G\left(y_{i}\right)\right)$ indicate the distribution of images $x_{i}$ and $G\left(y_{i}\right)$ , respectively. Here, the loss function of the generator and discriminator need to be updated. The discriminator “D” gets updated by the loss function:

$$-log\;D\left(x_{i}\right)-log\left(1-D\left(G\left(y_{i}\right)\right)\right)$$

and the generator “G” gets updated by the cost function, $MI\left(x_{i},G\left(y_{i}\right)\right)$ , where *G* is the generator and *D* is the discriminator, { $x_{i},y_{i}$ } is the training pair, *i* is the number of the image, H( $x_{i}$ ) is the entropy of image $x_{i}$ , and H( $x_{i}$ *, G(* $y_{i}$ *)*) is the joint entropy of these two images. By including joint entropy in the loss function, the amount of information in the output slice (generated image) was calculated based on the ground truth slice (real image). Moreover, the gradient descent optimizer (e.g., Adam) updates weights (model's parameters) in the direction of minimizing joint entropy via backward propagation. The loss function (joint histogram) value is low when images are aligned and high when images are not aligned. Therefore, the misalignment between the two images was implicitly fixed in this manner. This concept was proven in the registration framework using MI as the loss function.^37^, ^38^

#### 2.2.3 Discriminator

**Fig. 2:**
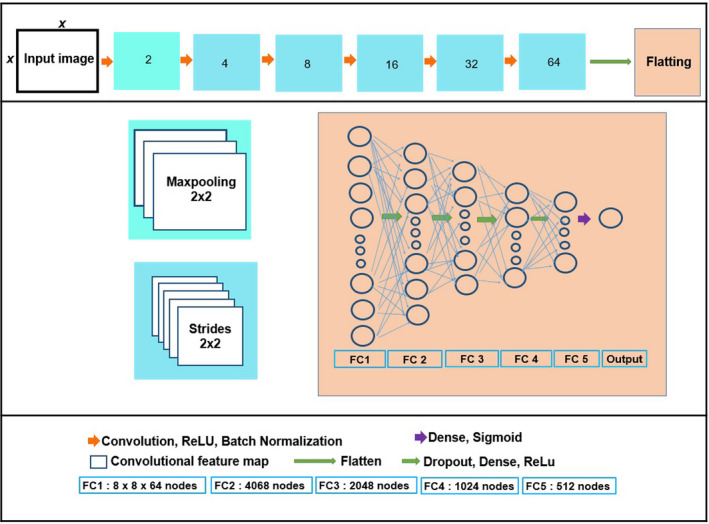
The structure of the discriminator with the details of convolutional and fully connected layers.

The discriminator consists of six convolutional layers, with different filter sizes, but the same kernel sizes and strides followed by five fully connected layers. The difference of our discriminator with the discriminator of conditional GAN model was adding more convolutional and fully connoted layers. The convolutional layers use ReLU as the activation function and include a batch normalization layer. The dropout layer was added to the fully connected layers, and a sigmoid activation function was used at the last fully connected layer. The binary cross entropy was employed for the loss function and Adam optimizer with learning rate = 0.00005, beta_1 = 0.5 in this network. We used 3x3 for the kernel size and 2, 4, 8, 16, 32, and 64 as the filter sizes in the discriminator network. The discriminator network takes an input with an image size of 352 × 352 and produces output as a real number in the range [0, 1]. The details of this structure are shown in Fig. 2.

### 2.3 Model training, validation, and testing

From a database of 77 patients, we randomly selected 66 patients (85%) for training and cross‐validation and used the remaining 11 patients (15%) for testing. The cross‐validation procedure, which is often used to evaluate the stability of the model,^39^ contained fivefolds in our case. For each fold, the set of 66 patients was divided into 54 patients (70%) for training and 12 patients (15%) for validation. The final model was selected as the best model from all folds (i.e., the one with the lowest loss function) and used to generate the sCT for the 11 test patients. The MRI/CT pairs used for training were non‐aligned, and thus, our aim was to overcome the difficulties related to MRI‐to‐CT registration by using the mutual information as the loss function (See Section 2.B.2). However, the data were paired, that is, each MRI and CT pair corresponded to the same patient. All operations were run on an NVIDIA TESLA K80 GPU with 12 GB dedicated RAM. We normalized our CT and MRI images by diving the value of each voxel to the maximum value of each image before training and testing our model. Then, in sCT images, we multiplied their voxels' values with maximum value of CT image and used the DICOM header information to go back to HU values in sCT images.

### 2.4 Treatment planning and dose calculation

The sCT was rigidly aligned to the CT using Eclipse software (Varian Medical Systems). Then, both the real and sCT images for each of the 11 patients in the test set were imported into the treatment planning system (RayStation v5.99 from RaySearch Laboratories AB). Target volumes and OARs—including left and right eye, optic nerves, optic chiasm, and brainstem—were contoured on MRI images. Then, the contours were transferred to registered CT images and finally reviewed by the radiation oncologist. In the next step, CT contours transferred to the registered sCT. An IMPT treatment plan was created on the CT, using robust (worst‐case) optimization for pencil beam scanning,^40^ with 3% range uncertainty and 3 mm systematic setup error, which led to 21 scenarios: nominal (zero shift) plus six shifts to the positive and negative directions in the DICOM axis (right–left, anterior–posterior, inferior–superior), times three range scenarios (0%, +3%, and −3%). The prescription dose to the target volume was 60 Gy. Two coplanar beams were used to create each plan, often placed in opposite directions (90º and 270º), or moved to more convenient angles when tumors were located in lateral–posterior positions. The IMPT plan was then used to recompute the dose on the sCT for dosimetric comparison, using the RayStation analytical (pencil beam) dose algorithm on a 3 × 3 × 3 mm^3^ grid. Analytical algorithms for proton therapy are known to have poor accuracy in the presence of tissue heterogeneities and range shifters.^28^, ^41^ Therefore, we used a second and independent Monte Carlo dose calculation engine, MCsquare,^42^, ^43^ to recompute the dose on both CT and sCT images, thus ensuring a proper analysis of the dosimetric accuracy of the sCT images. In addition, we performed a comprehensive robustness test, which included all treatment uncertainties (systematic, random setup errors, and range uncertainty), for each test patient in both CT and sCT using the MCsquare dose engine. This novel method for evaluating robustness combines all error parameters to simulate realistic treatment conditions, following a Monte Carlo approach.^44^ More specifically, the robustness test included 100 scenarios, each combining the effect of different treatment errors randomly sampled from their probability distribution as explained in the paper of Souris et al.^44^ The setup error values were sampled from a Gaussian distribution of σ = 1.2 mm in each direction (x, y, and z). The σ = 1.2 mm comes from Van Herk's margin recipe for a 3 mm margin (*m*) with a 90% confidence interval (Σ = 2.5) for the patient population, that is, *m* = Σσ^45^. The proton range errors were likewise sampled from a Gaussian distribution of σ = 1.6%, which was extracted from Paganetti’s guidelines for Monte Carlo dose calculations to achieve the recommended 2.4% range error (1.5 σ).^28^ The dose distribution was computed for each scenario using the fast Monte Carlo dose engine. The number of particles used for each simulation, around 10^7^ to 10^8^ particles, was adapted for each patient using a batch method so that the statistical noise remained below 2%.^46^ MCsquare used a dose grid resolution equal to the original size of the CT (0.68 × 0.68 × 1.50 mm^3^) for all computations, thus serving as an extra, high precision check on the dosimetric accuracy. The conversion from CT HU to stopping powers implemented in MCsquare is based on the Schneider stoichiometric calibration method.^47^ After dose calculation, the 10% of most extreme scenarios were disregarded in order to keep a 90% confidence level on the robustness evaluation, similar to the PTV concept used in conventional radiotherapy. A DVH‐band was generated for all ROI using the 90% selected scenarios. Then, the metrics corresponding to the worst‐case scenario (the borders of the DVH‐band) were evaluated as a measure of our plan quality and robustness for the treatment plans generated on both the CT and the sCT images. MCsquare used a dose grid resolution equal to the original size of the CT (0.68 × 0.68 × 1.50 mm^3^) for all computations, thus serving as an extra, high precision check on the dosimetric accuracy.

To assess the dosimetric impact of the HU differences between CT and sCT for all 11 test patients, we analyzed the dose volume histograms (DVHs) of both the pencil beam and the Monte Carlo doses computed on the two images. We also evaluated relevant metrics for the target volume and OARs, such as the mean dose (*D_mean_*) or the dose delivered to X% of the volume (*D_X_*). The robustness of each treatment plan on the CT and sCT images is analyzed and reported the results with DVH‐bands, from which the aforementioned metrics could be extracted for the nominal and worst‐case scenarios. In the robustness test, the DVHs corresponding to all 90 selected scenarios are plotted together in the form of DVH‐bands. Then, the metrics corresponding to the worst‐case scenario (the borders of the DVH‐band) are evaluated as a measure of our plan quality and robustness for the treatment plans generated on both the CT and the sCT images.

## 3 RESULTS

### 3.1 Synthetic CT generation

The proposed model generated the sCT in about 1 second per patient, and the training time for the model was around 33 h per fold during fivefold cross‐validation. To evaluate the accuracy of the generated sCT images, we calculated the mean absolute error (MAE) of the HU values for the whole external body contour (generated automatically in Eclipse for sCT and CT, respectively), for all patients in the test set. The average MAE ± SD (HU) values for all test data using each of the five models obtained after cross‐validation were 41.8 ± 10.0 (fold 1), 48.8 ± 13.0 (fold 2), 48.2 ± 12.4 (fold 3), 48.2 ± 12.2 (fold 4), and 48.3 ± 12.0 (fold 5). The average MAE over all cross‐validation sets was 47.2 ± 11.0.

### 3.2 Dosimetric evaluation

#### 3.2.1 Pencil beam dose (nominal case)

**Fig. 3:**
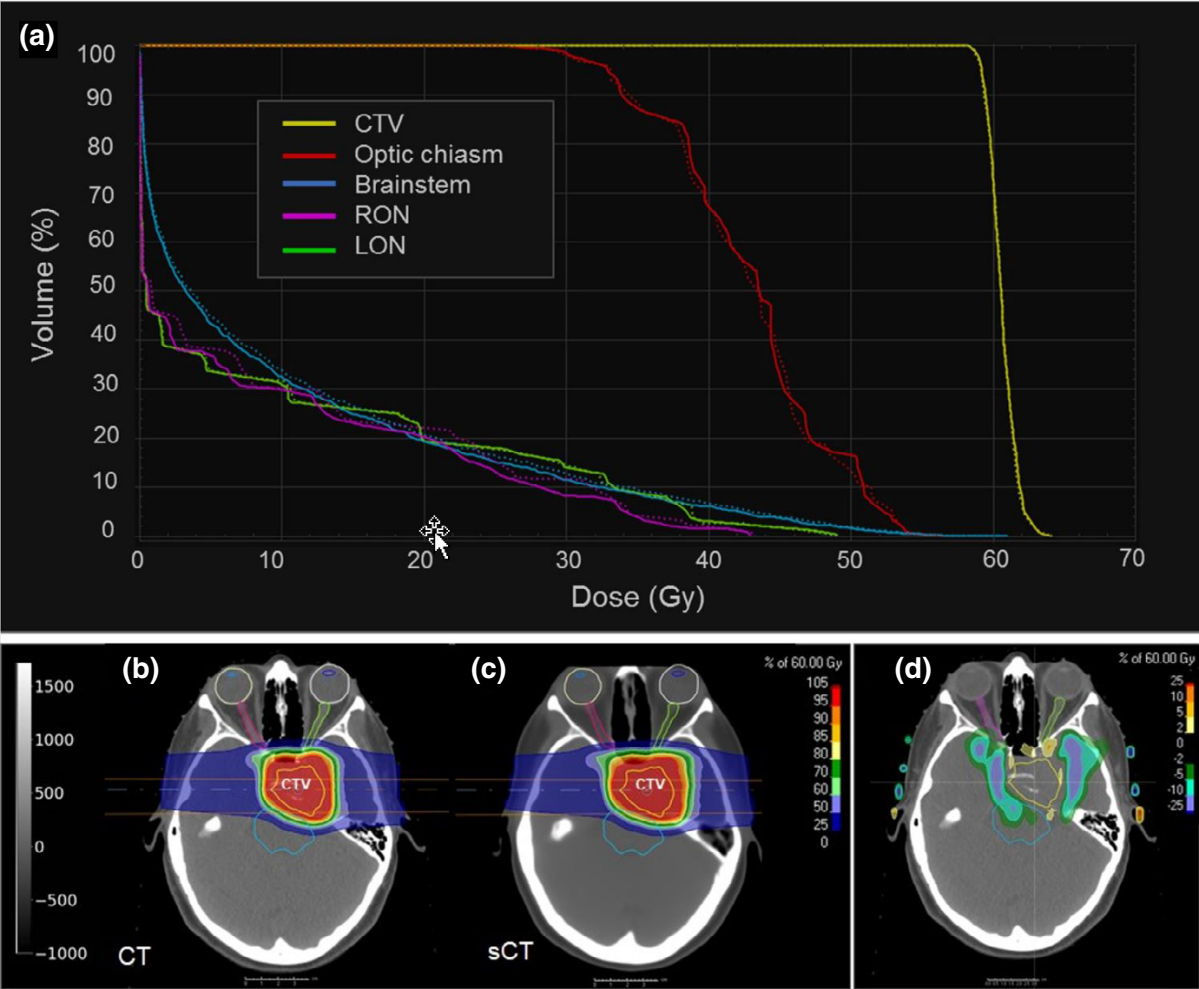
Dose volume histogram (a) for test patient #5 representing the pencil beam doses computed in computed tomography (CT) (solid line) and synthetic CT (dotted line) for the clinical target volume and the following organs at risks: right optic nerve, left optic nerve, brainstem, and optic chiasm. The corresponding dose distributions (at one slice in the center of the target) are presented in (b) for CT, (c) for sCT, and (d) the dose difference.

Table 1 presents the absolute differences between the DVH metrics, expressed as percentage (%) of the prescription dose, extracted from the nominal pencil beam doses computed by RayStation on the CT and sCT for all 11 test patients. The mean absolute difference for all metrics considered was below 2% (1.2 Gy). Indeed, most of the DVHs computed on CT and sCT overlapped except for some minor differences in specific cases. An example of the pencil beam doses for one of the test patients (patient #5) is presented in Fig. 3, together with the corresponding DVHs. The results for the CTV metrics, in particular, were remarkably similar, with differences below 0.5% (0.3 Gy) for both target coverage (D_95_ = 0.4 ± 0.4%) and overdose (D_5_ = 0.4 ± 04%). The difference was slightly higher for the metrics corresponding to the OARs we studied [left optic nerve (LON), right optic nerve (RON), brainstem, and optic chiasm], with an average difference in D_2_ ranging from 1% to 1.8%, and an average difference in D_mean_ ranging from 0.5% to 1.6%. Only a couple of patients reached a difference above 5% (3 Gy)—patient #1 (difference in brainstem D_2_ = 5.1% and RON D_2_ = 6.9%) and patient #10 (difference in optic chiasm D_2_ = 5.2% and D_mean_ = 6.0%), but these differences are not clinically relevant since the metric itself (D_2_) is far from the maximum dose that the organ can tolerate.

**Table 1:** Absolute differences between relevant dose volume histogram (DVH) metrics from the pencil beam doses (nominal case) computed on the computed tomography (CT) and synthetic CT for the 11 test patients, expressed as percentage (%) of the prescription dose (60 Gy). The last two columns contain the mean over all patients and its standard deviation (SD). NA: not applicable, when the organ was not contoured for a specific patient. LON/RON: left/right optic nerve.

Organ	D_X_	Patient number											Mean ± SD
		P1	P2	P3	P4	P5	P6	P7	P8	P9	P10	P11	
CTV	D_95_	1.1	0.2	0.5	0.2	0.1	0.2	0.9	0.5	0.2	0.1	0.9	0.4 ± 0.4
	D_5_	0.7	0.9	0.1	0.4	0.1	0.1	0.8	0.1	0.5	1.1	0.6	0.4 ± 0.4
	D_mean_	0.0	0.2	0.3	0.2	0.0	0.2	0.9	0.1	0.1	0.6	0.3	0.3 ± 0.3
Brainstem	D_2_	5.1	0.2	NA	0	0.6	0.7	1.0	0.4	0.8	0.2	1.1	1.0 ± 1.5
	D_mean_	1.1	1.0	NA	0	0.9	0.1	0.6	0.4	0.0	0.0	0.1	0.4 ± 0.4
Optic chiasm	D_2_	0.1	0.8	1.2	0	0.0	0	2.4	NA	0.2	5.2	NA	1.1 ± 1.7
	D_mean_	1.5	0.2	0.9	0	0.2	0	1.5	NA	0.6	6.0	NA	1.2 ± 1.9
LON	D_2_	2.4	0.0	3.2	0	0.5	0	2.5	0.3	0.4	0.4	NA	1.0 ± 1.2
	D_mean_	1.2	0.0	2.3	0	0.1	0	0.5	0.7	0.8	1.9	NA	0.7 ± 0.8
RON	D_2_	6.9	0.8	0.4	0	4.1	0	1.4	NA	0.5	NA	NA	1.8 ± 2.5
	D_mean_	1.5	0.2	0.0	0	1.2	0	0.0	NA	0.1	NA	NA	0.4 ± 0.6

#### 3.2.2 Monte Carlo dose (nominal case and robustness test)

**Fig. 4:**
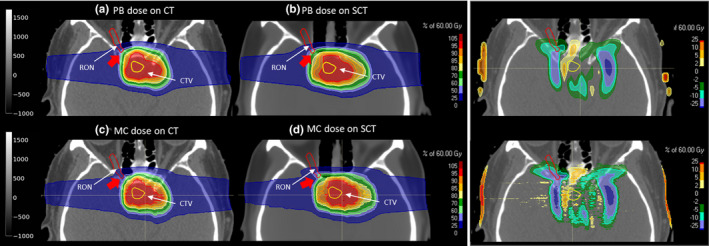
Pencil beam (PB) doses for patient #1 on the computed tomography (CT) (a) and synthetic CT (b), together with the dose difference, and the corresponding Monte Carlo (MC) doses (c and d). The right optic nerve (RON) and the clinical target volume are contoured in red and yellow, respectively. The red arrow points toward the region next to the nasal cavity, where a big discrepancy between pencil beam and MC doses in the sCT is found for the RON D_2_.

Table 2 presents the absolute differences between the DVH metrics obtained from the robustness test on the CT and sCT with the independent Monte Carlo dose engine (nominal and worst‐case scenarios) for all test patients, together with their means and standard deviations (SD). Again, the results for the CTV were particularly good and consistent with RayStation values for the nominal case, with mean differences for all metrics below 0.5%. The differences for the worst‐case metrics were slightly higher, especially for D_95_, which was slightly higher (worst D_95_ = 1.2 ± 1.5%). For this metric (D_95_), the difference between the worst case on CT and sCT for individual patients was above 2% (1.2 Gy) in a few cases (patients #1, #6, and #7), but it always remained under 5% (3 Gy). The MCsquare doses for the considered OARs presented a mean difference below 3% between all metrics computed on the CT and sCT for both nominal and worst cases. However, the mean difference in the worst case was around 0.2% to 1.5% higher than the difference in the nominal case. On the one hand, the differences in the nominal case given by the MC doses were mostly in agreement with the values extracted from RayStation, except for one case: patient #1, who presented a difference in D_2_ for RON equal to 17.9%, which was 11% higher than the value obtained from RayStation (Fig. 4). In this case, the affected organ (RON) has a very small volume and is close to the nasal cavity (Fig. 4), which increases the chance of the pencil beam algorithm providing a lower accuracy. On the other hand, the differences for the worst‐case scenario were below 3% on average, as previously reported, but again exceeded 5% in some exceptional cases, such as patient #1 (difference in worst‐case brainstem D_2_ = 9.8%, brainstem D_mean_ = 5.4%, LON D_2_ = 6.0%, and RON D_2_ = 14.8%), patient #2 (difference in worst‐case optic chiasm D_2_ = 12.1%), and patient #7 (difference in worst‐case brainstem D_2_ = 7.6%).

**Table 2:** Absolute differences between relevant dose volume histogram (DVH) metrics from the Monte Carlo doses (nominal and robustness test) computed on the computed tomography (CT) and synthetic CT for the 11 test patients, expressed as percentage (%) of the prescription dose (60 Gy). The values in regular font correspond to the nominal case, while those in *italics* correspond to the worst‐case scenario. The last two columns contain the mean over all patients and its standard deviation (SD). NA: not applicable, when the organ was not contoured for a specific patient. LON/RON: left/right optic nerve.

Organ	D_X_	Patient number											Mean *± *SD
		P1	P2	P3	P4	P5	P6	P7	P8	P9	P10	P11	
CTV	D_95_	1.3	0.8	0.2	0.4	0.2	0.5	0.5	0.0	0.1	0.7	0.3	0.5 *± 0.4*
		*2.8*	*0.0*	*0.2*	*0.2*	*0.6*	*2.2*	*4.7*	*0.6*	*0.2*	*1.0*	*0.8*	*1.2* *± 1.5*
	D_5_	0.0	0.0	0.2	0.3	0.5	0.2	0.7	0.3	0.0	0.3	0.4	0.3 *± 0.2*
		*0.2*	*0.2*	*0.1*	*0.5*	*0.3*	*0.1*	*0.5*	*0.7*	*0.0*	*0.4*	*0.4*	*0.3* *± 0.2*
	D_mean_	0.3	0.4	0.2	0.2	0.3	0.2	0.1	0.2	0.2	0.4	0.1	0.2 *± 0.1*
		*1.2*	*0.2*	*0.1*	*1.4*	*0.1*	*0.5*	*1.8*	*0.3*	*0.1*	*0.5*	*0.3*	*0.6* *± 0.6*
Brainstem	D_2_	5.1	0.1	NA	0.0	0.9	2.9	4.9	0.1	0.1	0.5	0.9	1.5 *± 2.0*
		*9.7*	*0.0*	*NA*	*0.0*	*2.0*	*2.3*	*7.6*	*2.4*	*1.7*	*1.4*	*2.6*	*3.0* *± 3.2*
	D_mean_	1.1	1.5	NA	0.0	0.8	0.2	0.5	0.6	0.2	0.1	0.3	0.5 *± 0.5*
		*5.4*	*2.3*	*NA*	*0.0*	*1.0*	*0.7*	*1.1*	*1.0*	*0.7*	*0.2*	*1.4*	*1.4* *± 1.5*
Optic chiasm	D_2_	0.3	1.4	0.5	0.0	0.7	0.0	1.9	NA	1.1	0.8	NA	0.7 *± 0.6*
		*0.6*	*12.1*	*0.5*	*0.0*	*1.1*	*0.0*	*2.5*	*NA*	*0.9*	*4.5*	*NA*	*2.5* *± 3.9*
	D_mean_	1.3	0.2	1.0	0.0	0.9	0.0	2.1	NA	0.2	0.7	NA	0.7 *± 0.7*
		*1.2*	*2.8*	*0.5*	*0.0*	*1.8*	*0.0*	*3.9*	*NA*	*0.2*	*1.0*	*NA*	*1.3* *± 1.4*
LON	D_2_	0.1	0.1	1.3	0.0	1.5	0.0	0.2	0.6	0.0	0.6	NA	0.4 *± 0.6*
		*6.0*	*0.1*	*0.6*	*0.0*	*0.9*	*0.0*	*2.3*	*1.2*	*0.8*	*3.6*	*NA*	*1.6* *± 1.9*
	D_mean_	1.3	0.0	2.4	0.0	0.1	0.0	0.6	0.7	0.3	3.1	NA	0.8 *± 1.1*
		*0.8*	*0.0*	*1.4*	*0.0*	*0.5*	*0.0*	*2.6*	*2.3*	*0.9*	*3.7*	*NA*	*1.2* *± 1.3*
RON	D_2_	17.9	0.7	0.0	0.0	2.4	0.0	1.0	NA	0.8	NA	NA	2.8 *± 6.1*
		*14.8*	*4.9*	*0.3*	*0.0*	*0.8*	*0.0*	*2.5*	*NA*	*0.5*	*NA*	*NA*	*3.0* *± 5.0*
	D_mean_	3.5	0.3	0.0	0.0	1.5	0.0	0.1	NA	0.4	NA	NA	0.7 *± 1.2*
		*3.1*	*1.5*	*0.1*	*0.0*	*1.5*	*0.0*	*1.5*	*NA*	*0.3*	*NA*	*NA*	*1.0* *± 1.1*

## 4 DISCUSSION

The analysis of the sCT images generated by our GAN model showed excellent agreement with the corresponding MRI images, with a very low difference in HU values. The mean absolute error (MAE) obtained over all test patients was 47.2 ± 11.0 HU, which is much lower than the values achieved in most previous studies using conventional sCT generation strategies, such as atlas‐based methods.^48^, ^49^ The MAE obtained is also slightly smaller than the values reported in recently published studies using deep learning methods, like the CNN method used by Dinkla et al.,^16^ who reported an MAE of 67 ± 11 HU, or the GAN model developed by Emami et al.,^50^ which achieved an MAE of 89.3 ± 10.3 HU. More recent publications from the group at Emory University used a three‐dimensional (3D) cycleGAN to generate sCT images, and obtained an MAE of 51.32 ± 16.91 HU for pelvic sCT^24^ and 72.87 ± 18.16 HU for liver sCT.^25^ A parallel work from Spadea et al.^26^ achieved an MAE value of 54 ± 7 HU for an sCT generation method for brain patients using a deep convolutional neural network (DCNN) model. Note that the comparison with published results from different groups can be affected by differences between patient datasets (tumor location, patient characteristics, etc.), which may influence reported HU errors. The novelty of this work is that it demonstrates the ability to use non‐aligned MR/CT pairs for training, which eliminates the need for rigid registration in the training MR/CT set. The use of mutual information in the generator’s loss function seems to be the key to overcoming issues related to non‐aligned images. Similar work^51^ used the conditional GAN architecture similar to the one presented here to generate sCT images and then to evaluate their photon‐based dosimetric accuracy for volumetric‐modulated arc therapy treatments. The mean percent difference between the doses calculated in CT and synthetic CT images was statistically insignificant and less than 1% overall for all DVH. The dosimetric results showed that the accuracy of the generated synthetic CT images was sufficient to produce clinically equivalent treatment plans. This previous work also compared the performance of a more conventional loss function based on MAE to the one including MI and showed the superiority of the MI‐based loss function (47.2 ± 11.0 HU error) over the MAE one (60.2 ± 22.0 HU error).

The data used for training were paired, that is, the MR/CT pairs were corresponding to the same patient. However, one of the advantages of GANs is the ability to learn from unpaired data. Learning image‐to‐image translation from unpaired data has achieved excellent results in fields like computer vision, but this task appears to be rather more complex when medical images are involved, since it requires the exact reproduction of the same patient anatomy, and not just any random or average patient anatomy. Nevertheless, it is an interesting topic to investigate in the future.

Besides generating accurate sCTs in terms of HU values, this work evaluated the dosimetric accuracy of the sCT images generated for scanned proton therapy treatment planning. For this purpose, robust IMPT plans were created on the CT images and recomputed on the sCT images for dosimetric comparison, using both the analytical pencil beam algorithm embedded in RayStation and the independent Monte Carlo dose engine MCsquare. In addition, we performed a comprehensive robustness test on the CT and sCT images using MCsquare to address the dosimetric accuracy of all possible uncertainty scenarios.

The results extracted from RayStation showed excellent agreement between CT and sCT images for most DVH metrics computed for the nominal case, with a mean absolute difference below 0.5% (0.3 Gy) of the prescription dose for the CTV and below 2% (1.2 Gy) for the OARs. This demonstrates a high dosimetric accuracy for the sCT images generated, especially in the target volume. Outside the target volume, the dosimetric accuracy decreases. The spatial dose differences were not performed for all the patients. However, from the visual inspection of the results, as illustrated in Figs. 3 and 4, the bigger dose differences happen in the edges of the CTV perpendicular to the beam direction. We believe that this is due to potential error. First, there is some level of registration error between CT and sCT. Second, the fact that the bone and air‐cavity regions are those regions where the model has the biggest prediction error. Thus, since the two opposite beams pass through the skull bone, the dose gets distorted (range undershoot/overshoot) due to the HU differences in this part. The superior accuracy obtained within the target volume may be explained by the use of robust optimization. In fact, only the objectives applied to the CTV and the brainstem were selected as robust, while the other OARs were treated as regular (non‐robust) volumes, that is, they were only evaluated in the nominal case during the optimization process. Selecting all organs as robust may help to increase the robustness against small HU variations for the rest of the organs and thus increase their dosimetric accuracy. However, this may increase the optimization time. Further investigation is needed to determine whether the dosimetric gain is worth the computational cost.

The metrics obtained from the Monte Carlo doses were mostly in agreement with the values extracted from RayStation for the nominal case (mean difference below 3%), which confirms the excellent dosimetric accuracy reported from the pencil beam doses. Only one case (patient #1) presented a large dose discrepancy for the right optic nerve (difference between CT and sCT in RON D_2_ equal to 17.9%, which was 11% higher than the value obtained from RayStation). In this particular case, the RON was very close to the nasal cavity, which is a challenging region for pencil beam algorithms because of the air, bone, and soft‐tissue interface. In addition, the volume of this structure is very small, which translates small point differences into big discrepancies for the associated dose metric (D_2_ in this case). Although this difference was not clinically relevant in our case because the dose was far below the clinical constraint, we recommend using an MC dose engine for final verification in cases where the organs are close to complex interfaces and the dose is close to an organ’s maximum tolerance. Note that the dose grid resolution used by MCsquare was equal to the original resolution of the CT and sCT (0.68 × 0.68 × 1.50 mm^3^), which is much smaller than the dose grid used in RayStation (3 × 3 × 3 mm^3^). This may also contribute to the differences seen when comparing the Monte Carlo and pencil beam results. Nevertheless, the final conclusions are drawn from the Monte Carlo results, which provide us with a very precise and accurate dosimetric evaluation.

For the worst‐case scenario, the differences between the doses computed on CT and sCT were slightly higher than for the nominal case in some patients, but they generally remained below 3%, except for a few metrics in certain patients (Table 2, patients #1, #2, and #7). Eventually, these errors could be reduced by increasing the robustness parameters used during plan optimization. In this study, we used 3 mm for the systematic setup error and 3% for the range uncertainty, which are the standard values reported in the literature. Increasing these values could help to reduce the sensitivity of the IMPT plans to the small differences in HU between the CT and sCT. But finding the most suitable values may require a detailed analysis of how best to translate the HU error associated with our sCT generation model into an equivalent robustness recipe, given the existing parameters available in commercial software (i.e., systematic setup error and constant range uncertainty). This type of study has already been performed to account for random setup errors,^52^ and a similar workflow could be applied to our particular problem. An alternative strategy to reduce the dosimetric differences between the CT and sCT would be to simulate HU errors directly in the robustness scenarios used during the optimization process. This would require generating an HU error distribution that could later be sampled to generate multiple scenarios to cover the entire error space.

As previously mentioned, the literature on the use of sCT images for MRI‐only proton therapy planning is rather scarce. However, given the increasing success of MR‐guided photon radiotherapy,^53^ we believe that the medical community will soon turn its attention to MRI‐guided proton therapy.^7^, ^54^ In fact, proton therapy patients could actually benefit even more than photon therapy patients from MRI‐only therapy planning, because the proton range's high sensitivity to tissue changes suggests an even greater need for adaptation and guidance during treatment. Additionally, several groups are investigating how to address the issues related to the behavior of charged particles (protons in this case) in a magnetic field, and they have achieved promising results.^55^, ^56^ Therefore, addressing the dosimetric accuracy of state‐of‐the‐art sCT generation methods for proton therapy, such as the one presented in this study, is crucial to bringing this technology closer to the clinic. So far, only few studies have evaluated the dosimetric accuracy of sCT images for proton therapy in brain patients. Rank et al.^57^ used a classification‐based tissue segmentation method to generate sCTs for three patients, which required two non‐standard sequences (ultrashort echo time [UTE] and turbo spin echo [TSE]), in addition to their regular protocol. They reported an MAE of 141–165 HU, with large deviations in air cavities and bones that led to underdosages to the target volume of up to 2%. Koivula et al.^20^ reported an MAE of 34 HU and a relative dose difference from sCT to CT within 0.5% in ten brain patients for their dual HU conversion model enabling heterogeneous tissue representation. However, their method excluded air cavity volumes, which is one of the most challenging parts, and required that the bone regions from the MRI images be segmented before the HU conversion. In both studies, the tumor was located in rather homogeneous regions, which might explain their good results, but they acknowledge the limitations of their method for tumors close to the nasal cavity, as is the case for some of our patients (Fig. 3). In addition, the need for multiple non‐standard MRI sequences or dedicated software for bone segmentation complicates the implementation of these methods in clinical practice. Another group analyzed the use of a commercial solution for creating bulk‐assigned sCTs for prostate patients^21^ and reported the need to manually adapt the assigned synthetic HU values by, for example, inserting the air cavities found on the CT. Again, the need for human intervention impedes the full automation of MRI‐only proton therapy planning and the implementation of MRI‐guided online treatment adaptation strategies.^7^ This is even more desirable for IMPT treatments than for conventional radiotherapy, given the potential to reduce inter‐ and intra‐fraction motion errors.^19^, ^58^ In contrast, the method proposed in this work enables a fast (1 s for sCT generation) and entirely automatic MRI‐only treatment planning process that removes all manual components from the workflow and achieves excellent dosimetric accuracy. A more recent study from Spadea et al.^26^ investigated the use of deep convolutional neural networks for sCT generation and also analyzed their dosimetric accuracy for single‐field uniform dose (SFUD) plans for brain tumor patients. In contrast, the present work investigated the dosimetric accuracy of the generated sCT for fully IMPT treatment planning, which is much more challenging than the case of SFUD due to the extra sensitivity of this technique to HU uncertainties. Therefore, worst‐case robust optimization on the CTV was used to generate the plans. Moreover, we performed a complete evaluation of the robustness of the generated plans, recomputing the dose on both CT and sCT for all considered uncertainty scenarios with an independent Monte Carlo dose engine. No previous study has performed such a complete dosimetric and robustness evaluation, which we believe is crucial for IMPT treatment plans, given their sensitivity to dose calculation and delivery uncertainties.

## 5 CONCLUSIONS

This work explanted the feasibility of using sCT images generated with a deep learning method based on generative adversarial networks (GANs) for intensity‐modulated proton therapy. We tested the method in brain tumors—some of them located close to complex bone, air, and soft‐tissue interfaces—and obtained excellent dosimetric accuracy even in those challenging cases. The proposed method can generate sCT images in around 1\,s without any manual pre‐ or post‐processing operations. This opens the door for online MRI‐guided adaptation strategies for IMPT, which would eliminate the dose burden issue of current adaptive CT‐based workflows, while providing the superior soft‐tissue contrast characteristic of MRI images.

## CONFLICT OF INTEREST

No conflict of interest.
